# Patient interactive digital support for women with adjuvant endocrine therapy in order to increase compliance and quality of life

**DOI:** 10.1007/s00520-020-05476-z

**Published:** 2020-05-13

**Authors:** Jenny Bergqvist, Staffan Lundström, Yvonne Wengström

**Affiliations:** 1 Breast Centre, Department of Surgery, Capio St Görans Hospital, Stockholm, Sweden; 2 Department of Oncology-Pathology, Karolinska Institutet, Stockholm, Sweden; 3 Palliative Care Services and R&D Department, Stockholms Sjukhem Foundation, Stockholm, Sweden; 4 Neurobiology Care Science and Society, Nursing, Karolinska Institutet, Stockholm, Sweden; 5 Cancer Theme, Breast Centre, Karolinska University Hospital, Stockholm, Sweden

**Keywords:** Breast cancer, Digital support, Patient reported outcome measures, Quality of life, Compliance, Adjuvant, Endocrine, Treatment

## Abstract

**Purpose:**

The primary aim of the study was to develop and investigate a patient interactive digital support (an app) for patients on adjuvant endocrine breast cancer treatment. Patient’s interactive digital applications are a fast-growing area for research and development. In general, patients want more information and support with regard to their diagnosis, treatment and self-care. At the same time, the health care system has limited resources for follow-up. Our primary endpoints were usability of the app and if it added any value to the patients.

**Methods:**

We designed and constructed a prototype, in dialogue with patients, containing four main modules for registration of drug compliance, performed physical exercise, self-care activities, and questions on health and quality of life. The app was then tested by patients and improved further before we completed a pilot study in which 15 patients used the app for 3 months.

**Results:**

Patients perceived the app easy to use with a very high median system usability score of 88.8, range 30–100. The 15 women registered in total 4251 times, range 118 to 372. The majority of registrations concerned compliance (adherence to treatment) and physical exercise.

**Conclusion:**

The app was perceived easy to use and of support in every-day life of breast cancer survivors. How to best integrate electronically collected patient reported outcome measures in clinical routine needs to be further studied, and future research will show if it will be cost-effective in terms of better health outcome and less resource use.

## Background

Breast cancer treatment is successful in the majority of cases due to the last decades’ development of new drugs. In addition to surgery, chemotherapy, antibody treatment and radiotherapy the adjuvant endocrine treatment follows for 5 to 10 years for the majority of women (80%). It is recommended for all with a hormone receptor positive breast cancer, reducing recurrent disease and breast cancer associated deaths [1].

We know from previous literature that many women stop to take their endocrine treatment before the recommended length of time. A retrospective study examining treatment adherence in over 780 breast cancer patients showed that non-adherence to radiation therapy, chemotherapy and endocrine therapy (tamoxifen) was 4%, 7% and 37% respectively [2].

According to a Swedish study 73–88% continued after 1 year, and only 50% carried on for all 5 years with endocrine treatment [1]. Two more retrospective studies, including 1451 and 8769 breast cancer patients showed close to 50% non-adherence to endocrine therapy [3, 4]. Registry research of prescribed drugs show that a lot of women with previous breast cancer do not use their prescription, without their doctors’ knowledge [5, 6].

Among the most important factors with impact on non-adherence to treatment are unmanageable side effects, patient-healthcare provider relationship, social support as well as depression and anxiety [7–9]. In addition, age, costs and perceived lack of support from the health care systems have been reported causes for ending treatment [1].

Patient interactive digital applications are a fast-growing area for research and development. In general, patients want more information and support with regard to their diagnosis, treatment and self-care. At the same time, the health care systems have limited resources for follow-up. Data indicate that adherence to adjuvant endocrine treatment can be improved by better support and education about breast cancer, treatment and self-care of side-effects [10, 11].

Our primary aim was to develop a patient interactive digital support for patients on adjuvant endocrine breast cancer treatment. Primary endpoints were usability and perceived value for patients in order to improve their quality of life and adherence to treatment.

## Material, patients and methods

The overall goal was to create a user-friendly patient interactive digital support which would add value to patients’ quality of life. We started with a focus group where three patients, all on adjuvant endocrine therapy, met to discuss their thoughts and associations to the words “digital support” and “endocrine therapy”. We developed our digital platform based on our previous experience of a well-documented design [12].

The projects´ three different phases:

1. Design and construction of a prototype in dialogue with three patients
2. Implementation and tests including tests by five patients
3. Pilot study with 15 patients. All patients received oral and written information and signed informed consent before they got access to the app and started registration of symptoms and quality of life for 3 months.

The app was co-created with patients diagnosed with breast cancer, who were involved throughout the development of the project. In phase 1, the focus was on design and functions of the app. Three women were interviewed in a focus group, asked about relevant problems and side effects to be assessed over time and what information they needed to engage in self-care activities. With their comments in mind and the ones from health care staff on an oncology department, about what questions patients usually came up with during their adjuvant endocrine treatment, we designed a prototype.

During phase 2, five patients tested the prototype and evaluated the usability and the information content in general. Their feedback was used for improvements before starting the pilot study including 15 patients, 41–78 years old women on adjuvant endocrine therapy, in phase 3 of the project. The primary endpoints of the trial were to investigate if the patients found the app easy to use and if it was of any value in their everyday life.

**Fig. 1:**
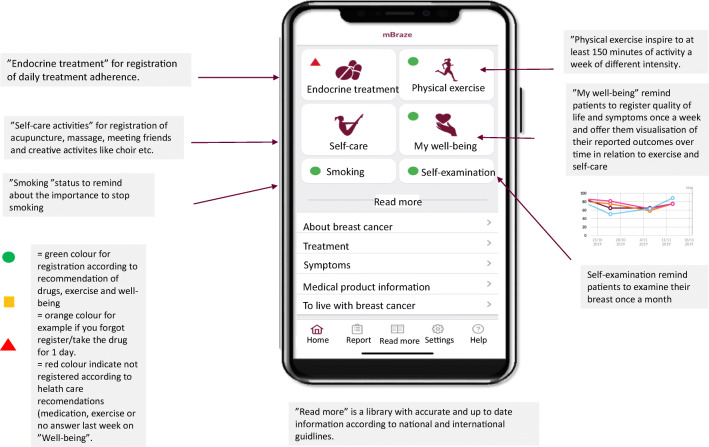
Patient interface of the application

The app contained four main modules including drug compliance, performed physical exercise, self-care activities, and questions on health and quality of life. In addition, it gave patients access to an extensive library with information about breast cancer, different treatment options, how to manage possible symptoms during treatment and support for coping with a breast cancer diagnosis. Another two modules on the front of the app reminded the women of self- examination of their breasts once a month and about their smoking habits. The interface of the app with four major and two minor modules in addition to an extensive library function is shown in Fig. 1.

In the pilot study, patients were encouraged to register information in the app about their physical exercise, information about self-care activities and if they had taken their adjuvant endocrine treatment for the day. They could if they preferred receive a daily reminder to take the medication. Once a week they were asked to answer questions about their health and quality of life in the section for “My well-being”. Patients could view their responses (patient reported outcome measures) over time and in relation to their registered physical exercise and self-care activities. Once a week, the app delivered an inspiring message about exercise or encouraged them to get in touch with health care if they had questions and or struggled with their medication or well-being.

The module “My well-being” contained 13 questions as follows: Were you limited in doing either your work or other daily activities? Were you limited in pursuing your hobbies or other leisure time activities? Were you limited in your sex-life? Did muscle and or joint pain interfere with your daily activities? Did you feel depressed? Have you had difficulty remembering things? Have you had difficulty in concentrating on things, like reading a newspaper or watching television? Did you have hot flushes? Did you have headaches? Did you have vaginal dryness? Have you had trouble sleeping? Have you felt physically less attractive as a result of your disease or treatment? Have you been dissatisfied with your body? The questions were extracted from the quality of life questionnaires EORTC QLQ-C30 and EORTC QLQ BR23.

At the end of the study, patients were asked to fill out the standardized System Usability Score (SUS) in order to investigate if the patients perceived the tool easy to use and if it added value [13]. In addition, they got a questionnaire with two questions: (1) What are your experiences from using the app during the study? (2) How did you perceive the use of the app? We also asked the women if they had any suggestions for improvement.

## Results

**Fig. 2:**
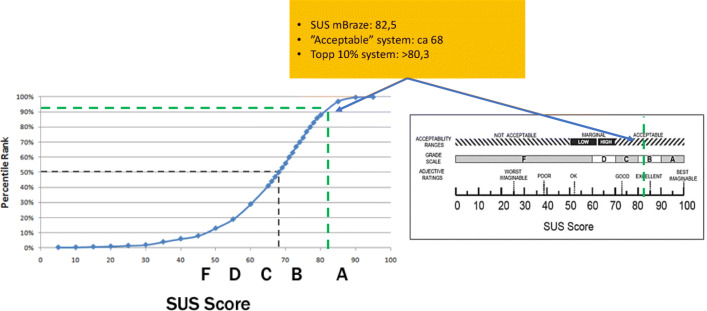
System usability score (SUS) of the study in comparison with literature: Usability.gov –Digital Communications Division in the U.S. Department of Health and Human Services’ (HHS) Office of the Assistant Secretary for Public Affairs; Bangor, A., Kortum, P. T., & Miller, J. T. (2009). Determining what individual SUS scores mean: Adding an adjective rating scale. Journal of Usability Studies, 4 [3], 114–123; Sauro, J. (2011). A practical guide to the System Usability Scale: Background, benchmarks, & best practices. Denver, CO: MeasuringUsability LLC

The median SUS was very high 88.8, range 30–100. One participant (who had problems with the internet connection at her summerhouse, which she perceived stressful, registered 30. Mean value of SUS 82.5. Evaluation of SUS in comparison with literature is displayed in Fig. 2.

**Fig. 3:**
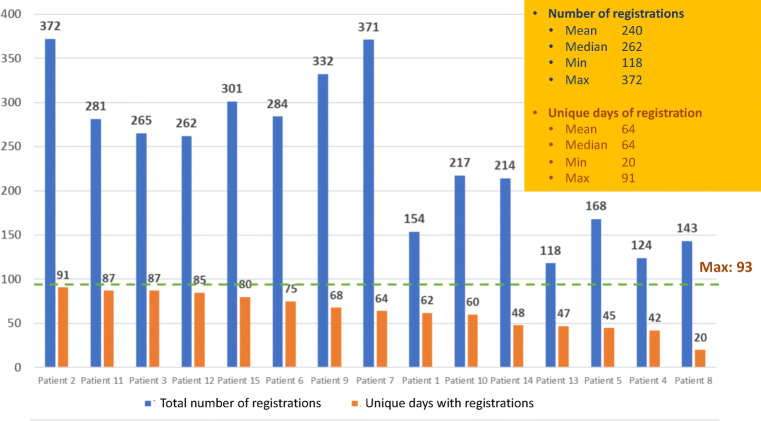
Total use of the digital application

There were in total 4251 registrations for the 15 participants, in median 262 registrations with a range from 118 to 372 for the 93 days (3 months) of the study. Unique days with registrations were in median 64, with a range from 20 to 64 days, Fig. 3.

The majority of registrations concerned treatment adherence (1582) and physical exercise (1125). There were 893 different registrations of self-care activities, of which the patients could register more than one/day.

**Fig. 4:**
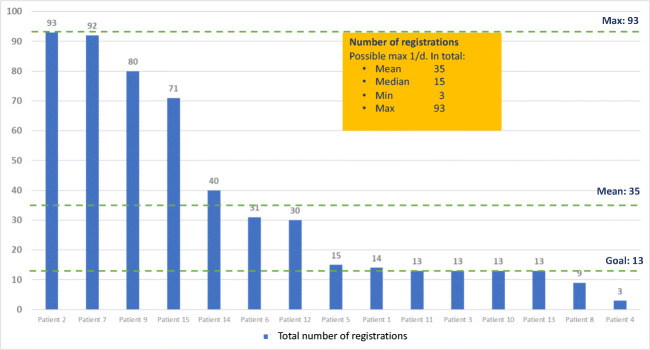
Registrations in module “My well-being”

Patients were encouraged to respond to the 13 questions in the module “My well-being” once a week. However, many patients registered their responses more often, in median 15 times, range 3–93 times. Registrations in the module of well-being are shown in Fig 4. All questions were answered by marking one of the four options, “Not at all”, “A bit”, Quite a bit” and “Very much”. The one only responding 3 times called to say she had no access to internet during her vacation, which was requested for this version of the app mBraze. Hot flushes, sleep disturbances and muscle and joint pain were the most disturbing symptoms according to the patient reported outcome measures in the module.

**Fig. 5:**
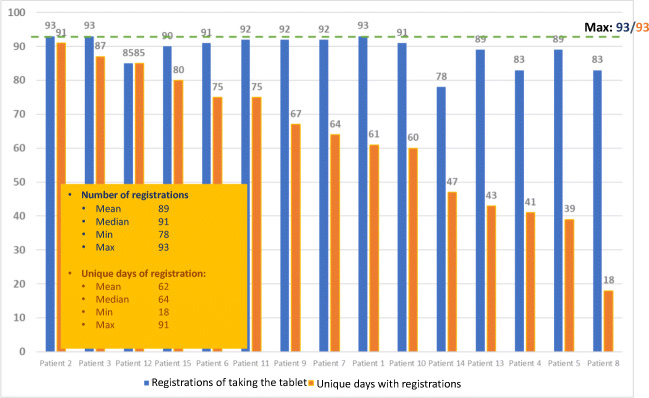
Registrations of drug-adherence in module “Endocrine therapy”

Almost all patients registered they had followed the recommended dose of one tablet a day for the 3 months of the study according to the module “Endocrine therapy”, Fig. 5. The majority registered once a day, but some chose to register afterwards for several days at a time.

**Fig. 6:**
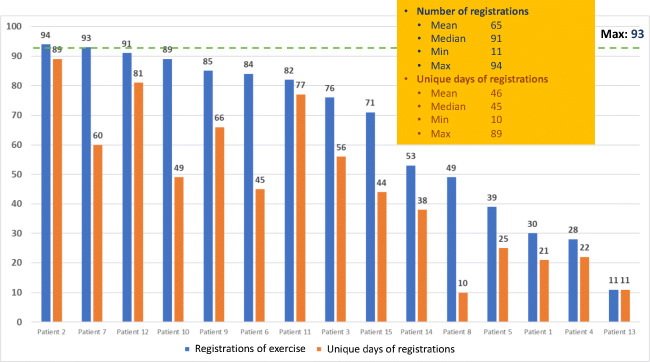
Registrations in module “Physical exercise”

In the module of physical exercise (Fig. 6), the patients were able to register for how many minutes they had been physically active during the day and if the activities were of low or high intensity. The patients had to be active for at least 150 min per week (WHO recommendation) to receive the green colour mark signalling well done for the week on the module for exercise (Fig. 1). Unique days of registration of physical exercise were in median 45, range 10–89. In mean, patients had walked 45 min per day.

**Fig. 7:**
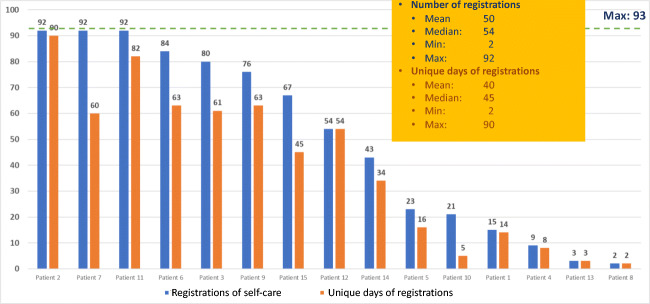
Registrations of “Self-care activities”

Figure 7 shows registrations of “Self-care activities” which were registered in median 54 times, range 2–92 times. Different actions for symptom relief and social activities were the most common self-care registered.

The qualitative part of the patients’ evaluation of the app showed that most patients wanted more functions. They wanted to be able to connect data from other applications to mBraze, like for example more advanced apps regarding exercise and food they ate. They also wanted to be able to connect to other patients using the app. In brief, they were all very satisfied with the visualization of their own symptom assessments over time, displayed in relation to their registered physical exercise and activity. Some quotes:

“A true wake up call, noticed I was not feeling well”

“I felt it helped me in preventing depression”

“Helped me in every-day life and to get back on track”

## Discussion

We developed a patient interactive digital support together with breast cancer patients with the aim to support and guide women with adjuvant endocrine treatment. Our pilot study showed the app was perceived easy to use and adding value in daily life for the 15 women included. SUS was in median very high (88.8). The women especially appreciated the possibility to overview their reported symptoms over time in relation to their activities and physical exercise. The women registered something at least every second day, and the majority said it had helped to motivate them to be more active and take better care of themselves.

Non-adherence to treatment is a concern for healthcare in general and treatment of breast cancer is no exception. Last years’ research report extended adjuvant endocrine therapy of 7–10 years to be in favour of 5 years for a majority of breast cancer patients [14]. The task to motivate patients to cope with extended adjuvant endocrine treatment will require even more health care recourses in the future. This is a challenge since health care has less time to spend on follow-up and support of breast cancer survivors in general. In a recently published Swedish study, including 21,016 patients with primary breast cancer, the 3 and 5 year adherence to endocrine therapy were 88% and 82,5%, respectively [15]. One of the most common causes of ending treatment is side effects like muscle and joint pain, estimated to affect approximately 50% of patients during treatment with aromatase inhibitors in clinical routine [16]. It often affects hands, wrists, knees, hips and feet and is associated with fatigue and insomnia [17]. Altogether, these symptoms have great negative impact on the quality of life of breast cancer survivors and may result in non-adherence to treatment. Another Swedish study of 1741 patients, all prescribed adjuvant endocrine therapy in 2005, reported a non-adherence to treatment of 31% after 3 years. Being married, younger age and larger tumour size were factors positively associated with adherence [18].

A limitation with our study is that it is a small pilot study for a relatively short period of time. The majority of patients had been prescribed adjuvant endocrine therapy for 1 year. Different needs of information and support may occur according to length of time since breast cancer diagnosis and the duration of endocrine therapy.

Previous studies indicate that electronic symptom screening in clinical routine may lead to increased symptom management [19] with better symptom control as a consequence of more frequent symptom discussions [20]. With better patient-health care relationship and communication the patients’ satisfaction, adherence to treatment and psychosocial adjustment will improve. Routine electronic collection of patient reported outcome measures might be one way to increase patient support and quality of life despite diminished resources for follow-up [21, 22].

Future randomised studies for longer periods of time are warranted with patient reported outcome measures in relation to resource use and health outcome of breast cancer care. Health economic studies still have to show if this is a way forward with e-health, which would improve both the quality of life for breast cancer survivors as well as make the breast cancer health care more cost efficient. Probably, personalised digital support with patient reported outcome measures online will be one way to improve patient-doctor interaction. It will be important that doctors and nurses will be able to take actions on the patient reported outcomes in due time. However, it must always be clear to patients that if any emergency occur they have to seek health care at the out-patient clinic or hospital.

We need economic evaluation and studies of patient safety before health care services may invest in tools for digital patient support. Future research will show if the cost for electronic devices and new systems for patient safety and follow-up will be outweighed by better healthcare outcome and less resource use [23, 24].

In conclusion, the app was perceived as easy to use and of support in every-day life of breast cancer survivors. How to integrate electronically collected patient reported outcome measures in clinical routine for best patient support, needs to be further studied and should be on the top agenda for health care services.

## References

[1] Chlebowski RT, Kim J, Haque R (2014). Adherence to endocrine therapy in breast cancer adjuvant and prevention settings. Cancer Prev Res (Phila).

[2] Ma AM, Barone J, Wallis AE (2008). Noncompliance with adjuvant radiation, chemotherapy, or hormonal therapy in breast cancer patients. Am J Surg.

[3] Hershman DL, Kushi LH, Shao T, Buono D, Kershenbaum A, Tsai WY, Fehrenbacher L, Lin Gomez S, Miles S, Neugut AI (2010). Early discontinuation and nonadherence to adjuvant hormonal therapy in a cohort of 8,769 early-stage breast cancer patients. J Clin Oncol.

[4] van Herk-Sukel MP, van de Poll-Franse LV, Voogd AC, Nieuwenhuijzen GA, Coebergh JW, Herings RM (2010). Half of breast cancer patients discontinue tamoxifen and any endocrine treatment before the end of the recommended treatment period of 5 years: a population-based analysis. Breast Cancer Res Treat.

[5] He W, Fang F, Varnum C, Eriksson M, Hall P, Czene K (2015). Predictors of discontinuation of adjuvant hormone therapy in patients with breast cancer. J Clin Oncol.

[6] Ziller V, Kalder M, Albert US, Holzhauer W, Ziller M, Wagner U, Hadji P (2009). Adherence to adjuvant endocrine therapy in postmenopausal women with breast cancer. Ann Oncol.

[7] Paranjpe R, John G, Trivedi M, Abughosh S (2018) Identifying adherence barriers to oral endocrine therapy among breast cancer survivors. Breast Cancer Res Treat 174(2):297–305 10.1007/s10549-018-05073-z 30523459

[8] DiMatteo MR, Lepper HS, Croghan TW (2000) Depression is a risk factor for noncompliance with medical treatment: meta-analysis of the effects of anxiety and depression on patient adherence. Arch Intern Med 160:2101–2107 10.1001/archinte.160.14.2101 10904452

[9] Lambert LK, Balneaves LG, Howard AF, Gotay CC (2018) Patient-reported factors associated with adherence to adjuvant endocrine therapy after breast cancer: an integrative review. Breast Cancer Res Treat 167:615–633 10.1007/s10549-017-4561-5 29110151

[10] Regional cancer centres in cooperation in Sweden (2018) National guidelines of breast cancer care

[11] Group* SBCC (1996) Randomized trial of two versus five years of adjuvant tamoxifen for postmenopausal early stage breast cancer. J Natl Cancer Inst 88(21):1543–1549 10.1093/jnci/88.21.1543 8901852

[12] Johnston N, Bodegard J, Jerstrom S et al (2016) Effects of interactive patient smartphone support app on drug adherence and lifestyle changes in myocardial infarction patients: a randomized study. Am Heart J 178:85–94 10.1016/j.ahj.2016.05.005 27502855

[13] Bangor A, Kortum P, Miller J (2009) Determining what individual SUS scores mean: adding an adjective rating scale. J Usability Stud 4:114–123

[14] Gea C (2017) De-escalating and escalating treatments for early-stage brest cancer. The St Gallen international expert consensus conference on the primary therapy of early breast cancer. Ann Oncol 1700–1712 10.1093/annonc/mdx308 PMC6246241 28838210

[15] Andersson A, Väppling A, Von Wachenfeldt, De Jong A, Nyström L (2019) Adherence to adjuvant endocrine therapy after breast cancer in Sweden 2008–2010: A nationwide survey. Am Soc Clin Oncol. 10.1200/JCO.2019.37.15

[16] Crew KD, Greenlee H, Capodice J, Raptis G, Brafman L, Fuentes D, Sierra A, Hershman DL (2007). Prevalence of joint symptoms in postmenopausal women taking aromatase inhibitors for early-stage breast cancer. J Clin Oncol.

[17] Bauml J, Chen L, Chen J, Boyer J, Kalos M, Li SQ, DeMichele A, Mao JJ (2015). Arthralgia among women taking aromatase inhibitors: is there a shared inflammatory mechanism with co-morbid fatigue and insomnia?. Breast Cancer Res.

[18] Wigertz A, Ahlgren J, Holmqvist M, Fornander T, Adolfsson J, Lindman H, Bergkvist L, Lambe M (2012). Adherence and discontinuation of adjuvant hormonal therapy in breast cancer patients: a population-based study. Breast Cancer Res Treat.

[19] Seow H, Sussman J, Martelli-Reid L, Pond G, Bainbridge D (2012). Do high symptom scores trigger clinical actions? An audit after implementing electronic symptom screening. J Oncol Pract.

[20] Detmar SB, Muller MJ, Schornagel JH, Wever LD, Aaronson NK (2002). Health-related quality-of-life assessments and patient-physician communication: a randomized controlled trial. JAMA.

[21] Blanch-Hartigan D, Chawla N, Moser RP, Finney Rutten LJ, Hesse BW, Arora NK (2016). Trends in cancer survivors' experience of patient-centered communication: results from the health information National Trends Survey (HINTS). J Cancer Surviv.

[22] Moore PM, Rivera S, Bravo-Soto GA, Olivares C, Lawrie TA (2018). Communication skills training for healthcare professionals working with people who have cancer. Cochrane Database Syst Rev.

[23] Chen J, Ou L, Hollis SJ (2013). A systematic review of the impact of routine collection of patient reported outcome measures on patients, providers and health organisations in an oncologic setting. BMC Health Serv Res.

[24] Mayer DK, Travers D, Wyss A, Leak A, Waller A (2011). Why do patients with cancer visit emergency departments? Results of a 2008 population study in North Carolina. J Clin Oncol.

